# Supplementary material for: Microbial and metabolic succession on common building materials under high humidity conditions
Source: Nat Commun. 2019 Apr 16;10:1767. doi: 10.1038/s41467-019-09764-z (PMC6467912; doi:10.1038/s41467-019-09764-z)
Supplement: Supplementary file 1 — Supplementary Information [file 41467_2019_9764_MOESM1_ESM.pdf]

SUPPLEMENTARY INFORMATION

FOR

Microbial and Metabolic Succession on Common Building Materials Under High Humidity Conditions

Lax *et al.*

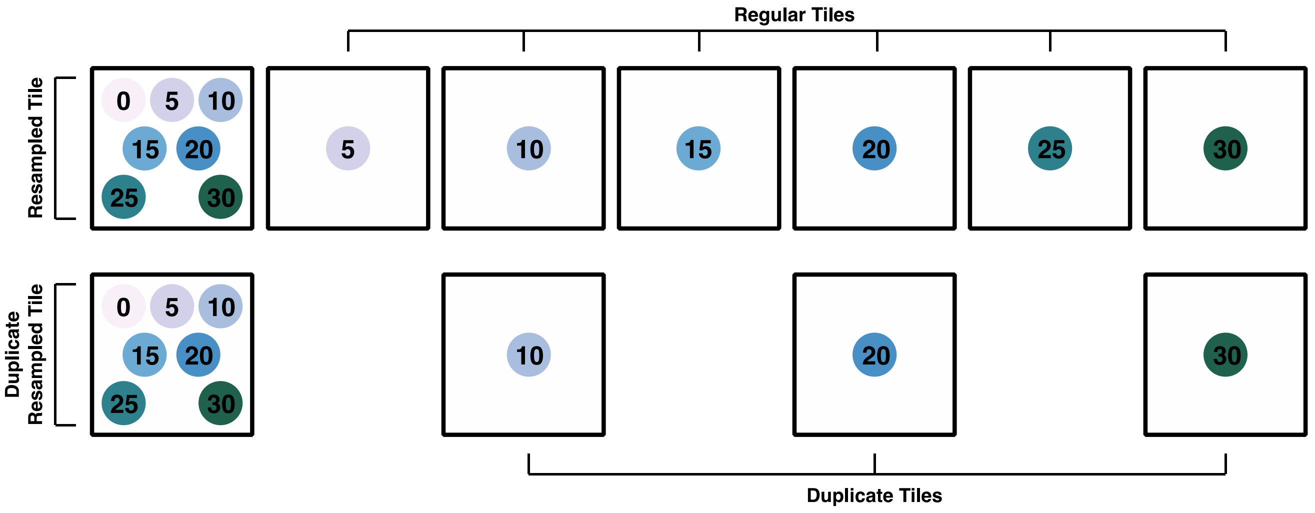

Figure S1: Experimental setup of project. Illustration of the experimental setup and coupon sampling procedures**.** Numbers indicate the day of study on which the tile was sampled.

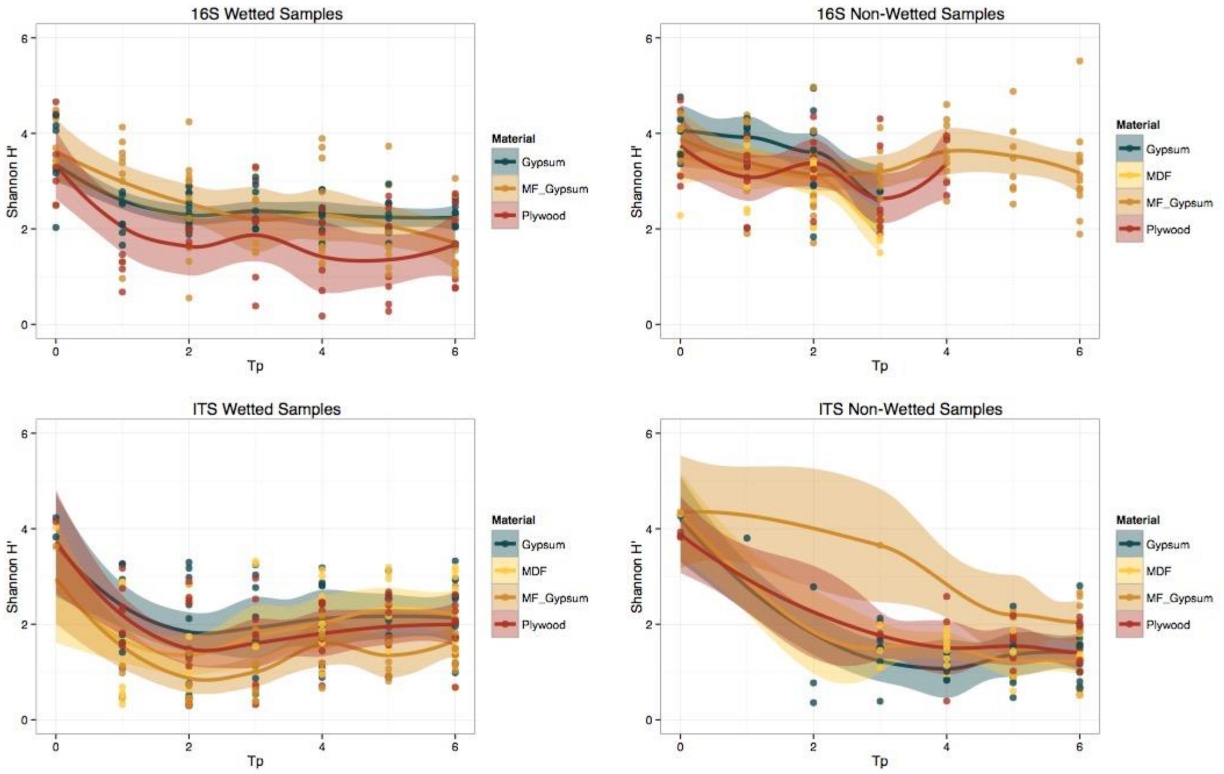

Figure S2: Change in the Shannon Index by material over time. Points represent individual samples (n=338, 330 samples for 16S and ITS, respectively) and the trend lines are a smoothed moving-average of the mean and shaded regions indicate the standard error

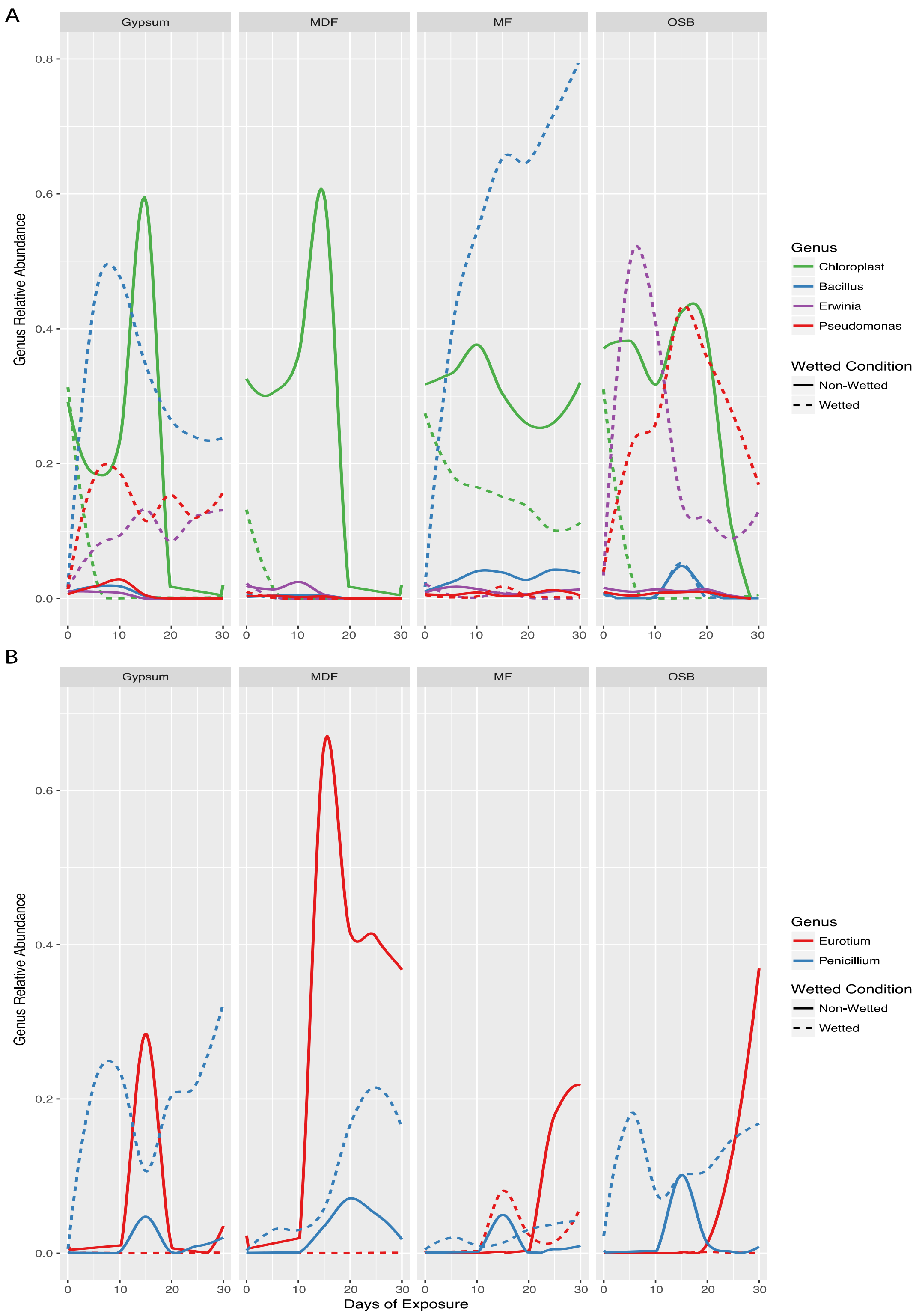

Figure S3: Microbial succession by material over time Changes in the relative abundance of selected microbial genera for each material over the course of succession. (n=338, 330 samples for 16S and ITS, respectively). (A) Lines represent a smoothed moving average of the mean. Genus is indicated by color and wetting condition is indicted by line style. (**B**) Changes in the relative abundance of selected fungal genera over the course of succession. Formatting is as in (**A**)

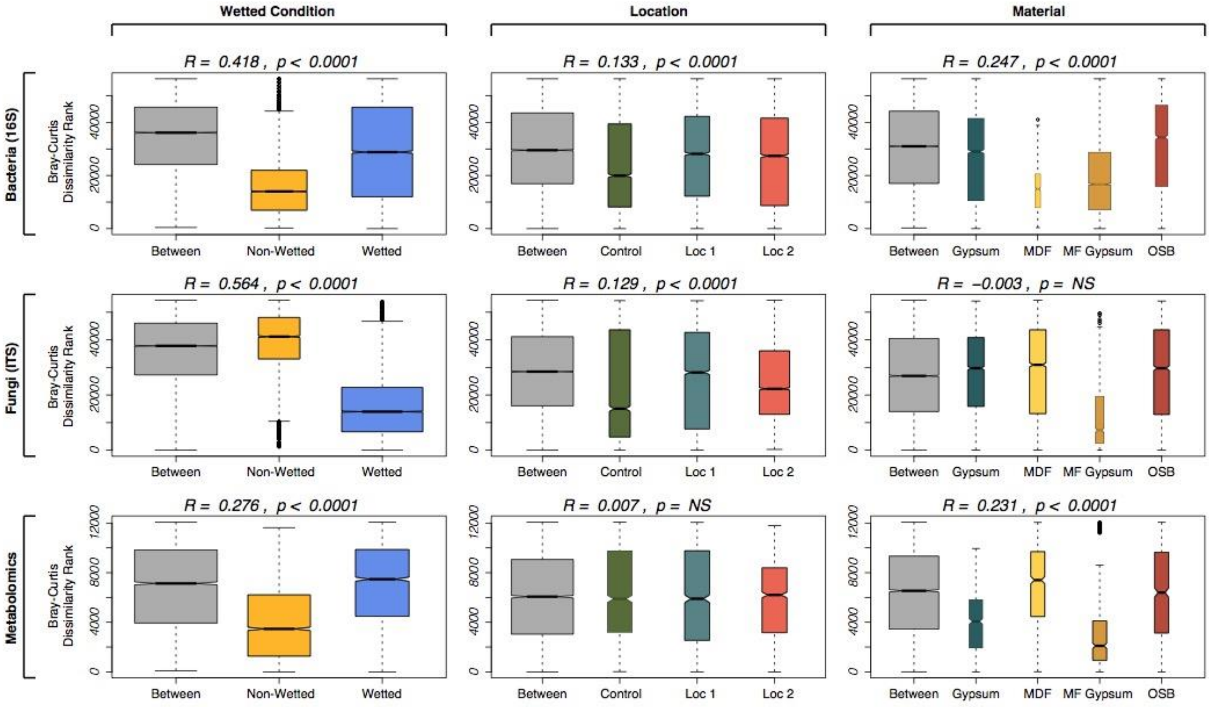

Figure S4: ANOSIM quantifies the influence of metadata factors on the dissimilarity between samples. Columns represent different metadata factors and rows represent the three datasets in this study (n=338, 330 samples for 16S and ITS, respectively). Boxplots depict the range of ranked Bray-Curtis dissimilarities within and between factors (lower rank = lower dissimilarity). Boxplot width indicates the number of samples represented by the boxplot

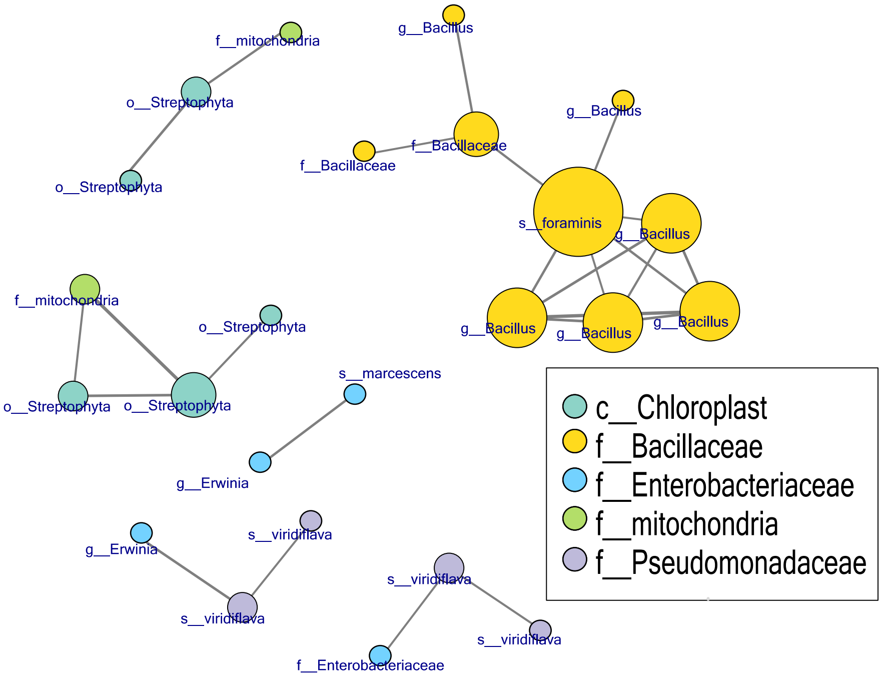

Figure S5: Bacteria-Bacteria co-occurrence network. Co-occurrence network (from n=83 bacteria samples) shows highly correlated bacteria form monophyletic clusters for samples containing more abundant taxa

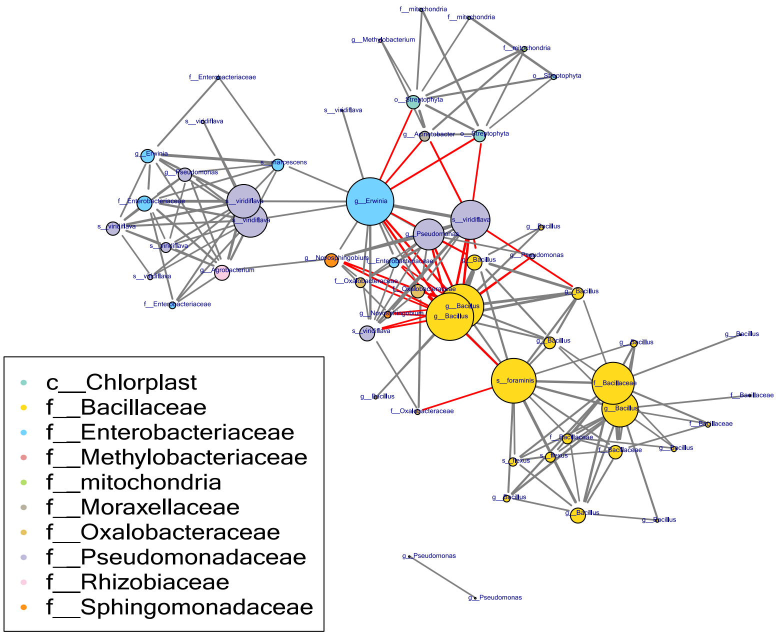

Figure S6: Bacteria-Bacteria co-occurrence network on wet samples. Co-occurrence network (from n=39 wet samples) shows how *Pseudomonas* and *Bacillus* are anticorrelated on wet samples for samples containing more abundant taxa

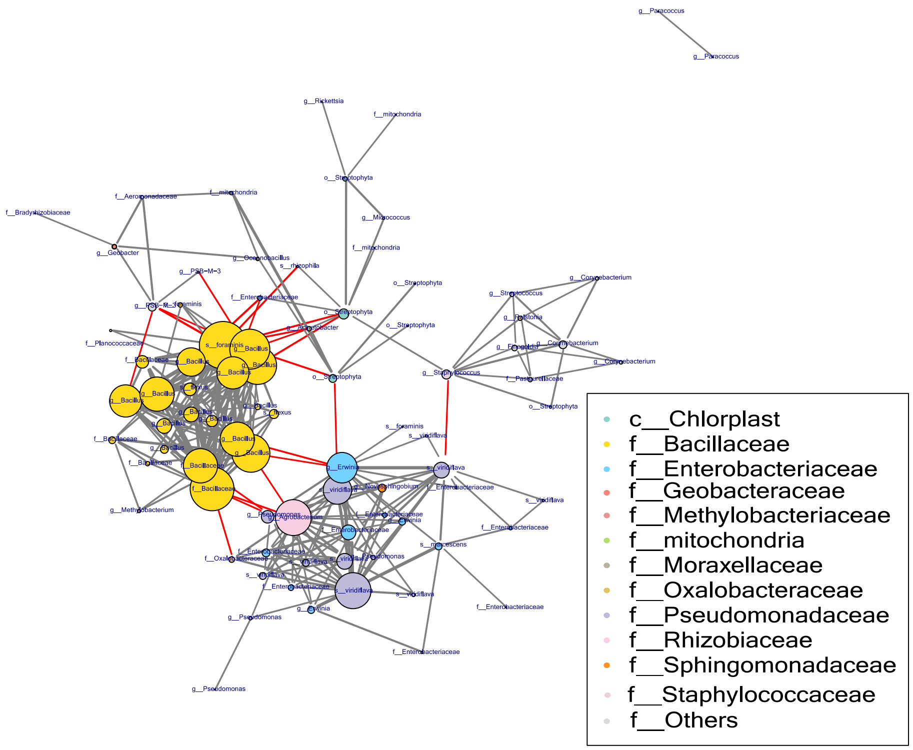

Figure S7: Bacteria-Bacteria co-occurrence network on gypsum samples. Co-occurrence network (from n=24 gypsum samples) shows how *Pseudomonas* and *Bacillus* are anticorrelated on all gypsum samples

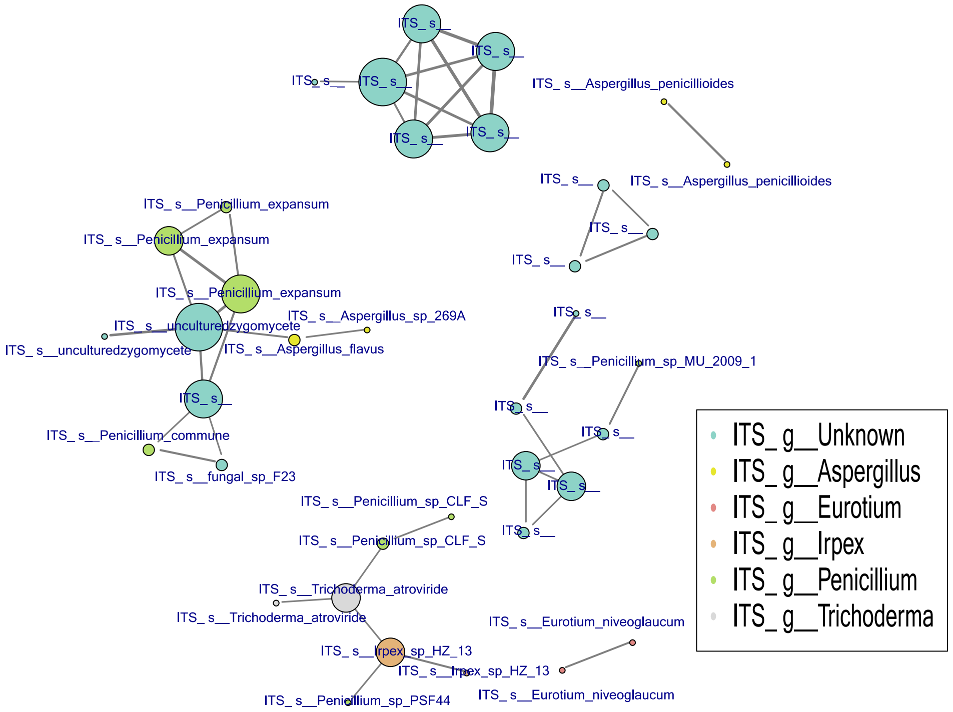

Figure S8: Fungi-Fungi co-occurrence network. Co-occurrence network (from n=91 fungi samples) shows highly correlated fungi forms mostly monophyletic clusters

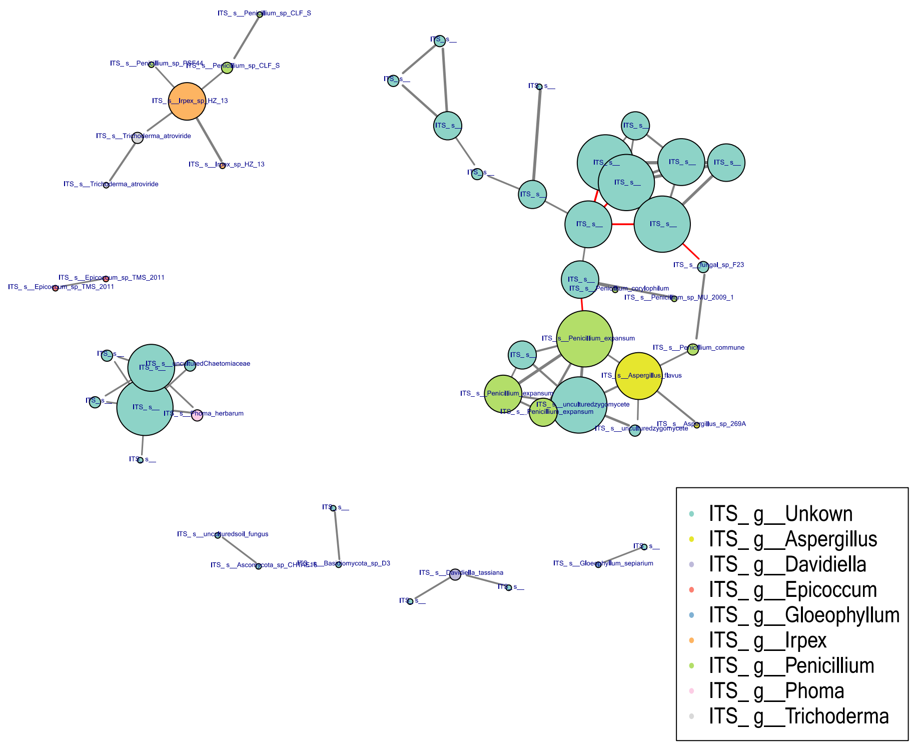

Figure S9: Fungi-Fungi co-occurrence network on wet samples. Co-occurrence network (from n=58 wet samples) shows how certain Fungi OTUs are anticorrelated on wet samples

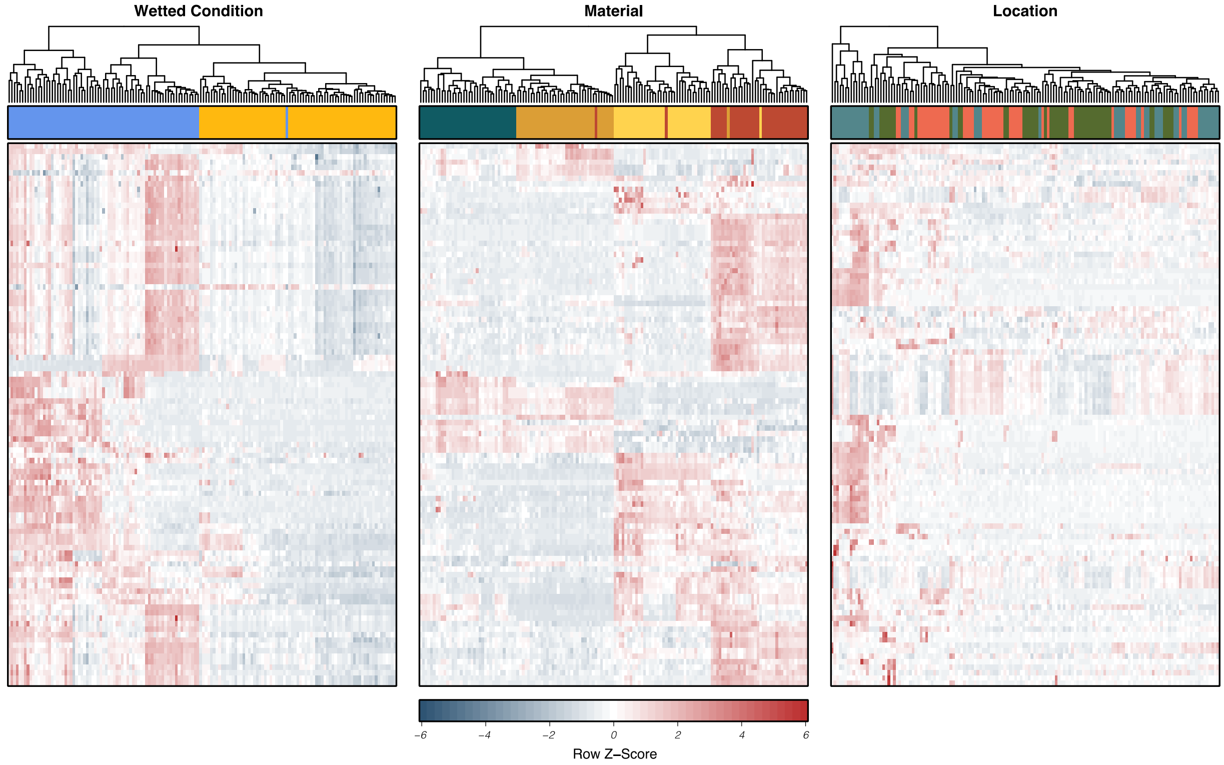

Figure S10: Random forest metabolite selection heatmap. Random forest learning was used to select the metabolites that most distinctly identify each environmental condition, wetted or non-wetted, wood material type and inoculation location (n=144 samples and 3187 metabolites), the 100 highest-scoring metabolite features for each condition where selected for further examination.

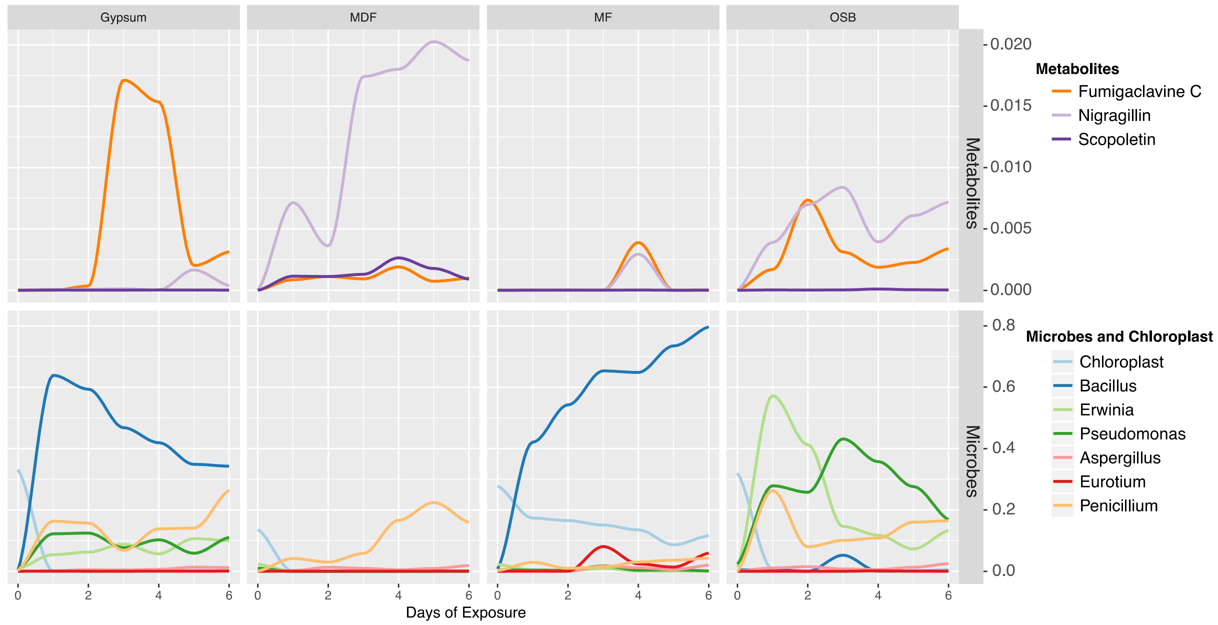

Figure S11: Metabolite and microbial succession on wet samples by material over time. Changes in the relative abundance of selected bacterial genera for each material over the course of succession (n=176, 218 and 72 wet samples for bacteria, fungi and metabolites, respectively). Lines represent a smoothed moving average of the mean. Genus and metabolites are indicated by different colors

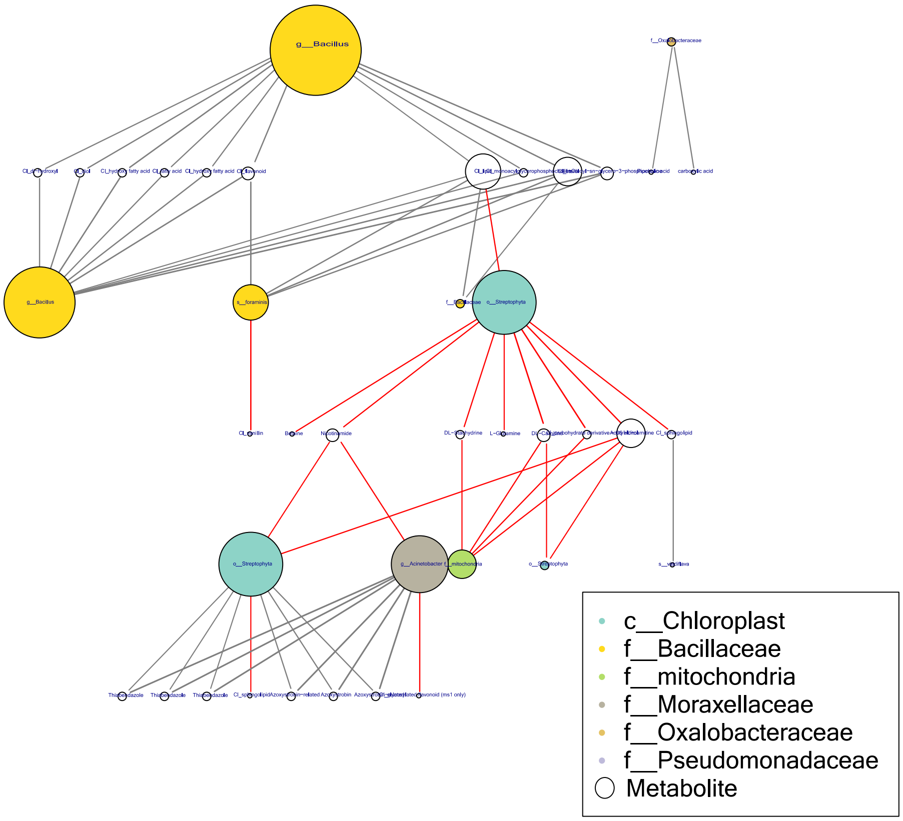

Figure S12: Bacteria-Metabolite co-occurrence network. Bacteria and Metabolite paired co-occurrences suggesting biochemical exchanges (from n=83, 144 samples, respectively). Lipid and hydroxyl compounds are strongly connected to *Bacillaceae* groups. Some specific lipids correlate positively with bacteria and negatively with wood material (plants). Vitamins and small carbon compounds negatively correlate with the wood material (plants).

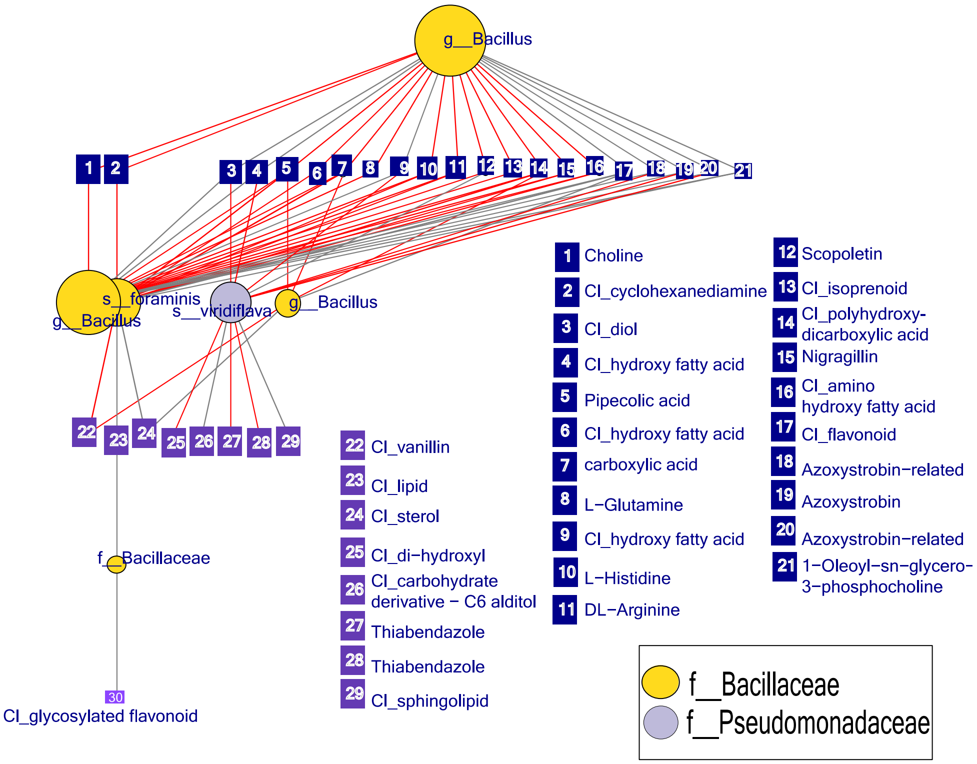

Figure S13: Bacteria-Metabolite co-occurrence network for Bacillus and Pseudomonas interactions only. Bacteria and Metabolite paired co-occurrences suggesting biochemical exchanges (from n=83, 144 samples, respectively). *Nigragillin* is negatively correlated with both *Pseudomonas* and *Bacillus*. *Azoxystrobin* correlates negatively with *Pseudomonas*, but positively with *Bacillus*. Hydroxyl compounds correlates negatively with *Pseudomonas* but positively with *Bacillus*

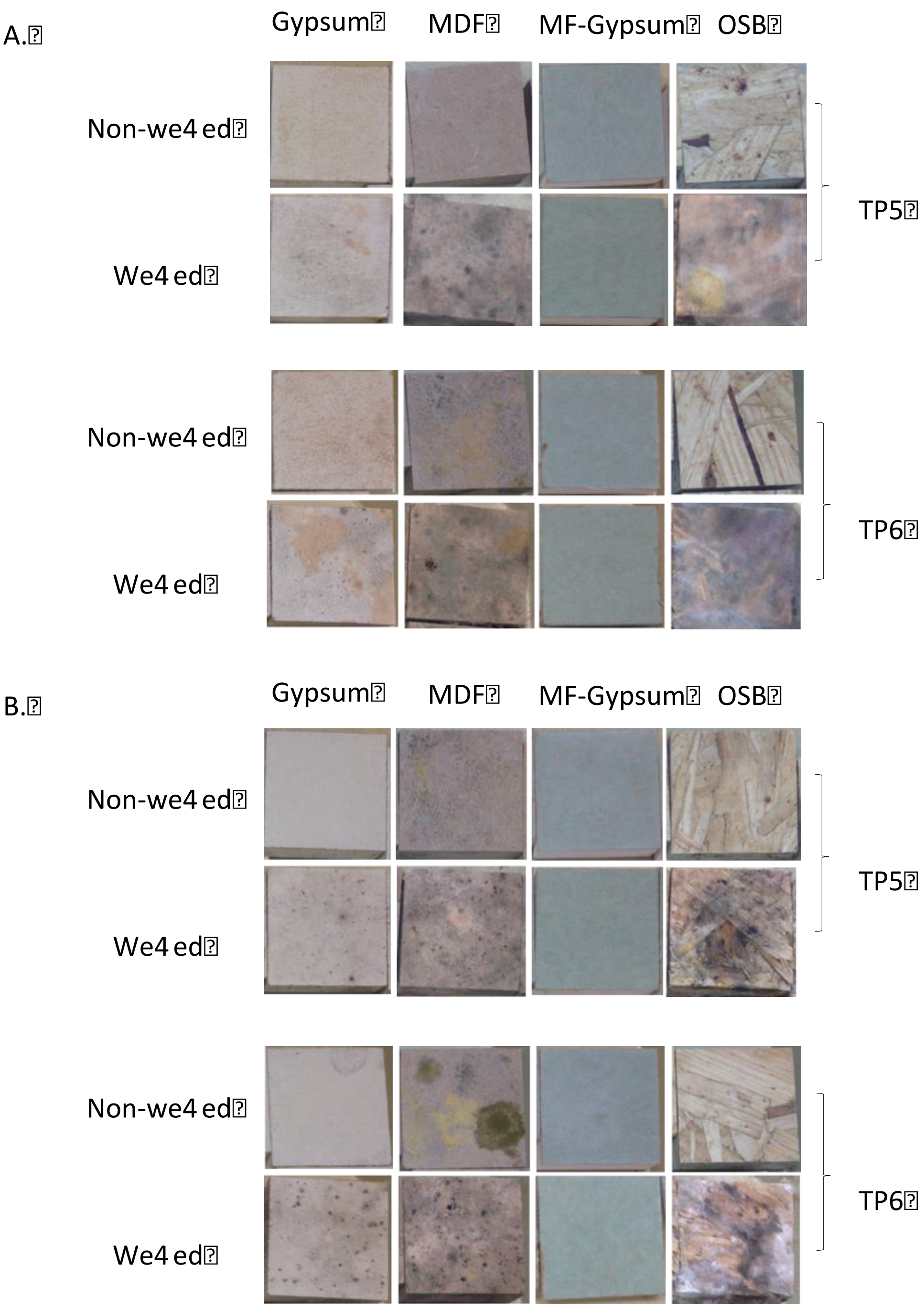
A.
Gypsum
MDF
MF-Gypsum
OSB
Non-we4 ed
We4 ed
TP5
Non-we4 ed
We4 ed
TP6
B.
Gypsum
MDF
MF-Gypsum
OSB
Non-we4 ed
We4 ed
TP5
Non-we4 ed
We4 ed
TP6

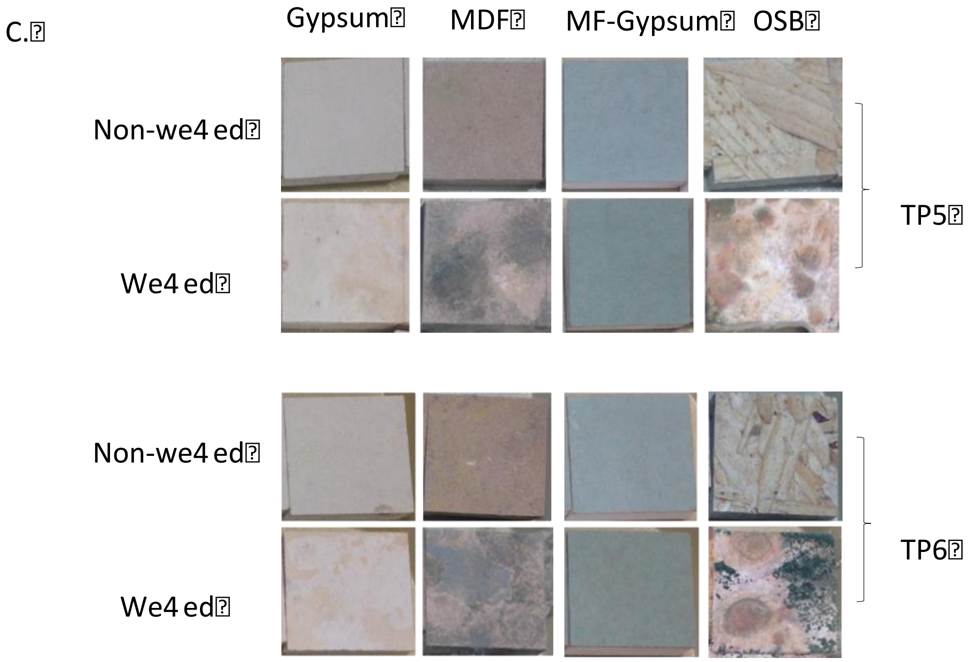

Figure S14: Photographs of wood coupons from different materials and wetting conditions at TP5 and TP6. Bacterial and Fungal growth on coupons surface photographs for (A) location 1 (B) location 2 and (C) control location.

	Bacteria (16S)		Fungi (ITS)		Metabolomics	
	R^2	*p*	R^2	*p*	R^2	*p*
Wetted	0.374	*< 0.0001*	0.450	*< 0.0001*	0.204	*< 0.0001*
Timepoint	0.130	*< 0.0001*	0.094	*< 0.0001*	0.178	*< 0.0001*
Control	0.074	*< 0.0001*	0.107	*< 0.0001*	0.001	*0.983*
Location 1	0.045	*0.0006*	0.076	*0.0006*	0.009	*0.502*
Location 2	0.214	*< 0.0001*	0.303	*< 0.0001*	0.007	*0.603*
Gypsum	0.085	*< 0.0001*	0.022	*0.0271*	0.141	*<0.0001*
MDF	0.108	*< 0.0001*	0.009	*0.2399*	0.238	*<0.0001*
MF Gypsum	0.027	*0.0095*	0.008	*0.2720*	0.209	*<0.0001*
OSB	0.041	*0.0015*	0.002	*0.762*	0.503	*<0.0001*
Bacillus	0.259	*< 0.0001*	*NA*	*NA*	*NA*	*NA*
Pseudomonas	0.088	*< 0.0001*	*NA*	*NA*	*NA*	*NA*
Erwinia	0.098	*< 0.0001*	*NA*	*NA*	*NA*	*NA*
Chloroplast	0.196	*< 0.0001*	*NA*	*NA*	*NA*	*NA*
Penicillium	*NA*	*NA*	0.106	*<0.0001*	*NA*	*NA*
Eurotium	*NA*	*NA*	0.185	*<0.0001*	*NA*	*NA*

Table S1: ANOSIM results calculate the factors significantly correlated with differences in the microbial communities across our three datasets, Bacteria, fungi, Metabolomics

	REG			RE			RE.DUP			DUP		
	mantel	significance	n	mantel	significance	n	mantel	significance	n	mantel	significance	n
REG	<NA>			0.67	1E-05	74	0.71	1E-05	79	0.81	1E-05	44
RE	0.67	1E-05	74	<NA>			0.85	1E-05	77	0.75	1E-05	40
RE.DUP	0.71	1E-05	79	0.85	1E-05	77	<NA>			0.79	1E-05	39
DUP	0.81	1E-05	44	0.75	1E-05	40	0.79	1E-05	39	<NA>		

Table S2: Mantel test results calculate the correlation among fungi samples across different sampling strategies.

	REG			RE			RE.DUP			DUP		
	mantel	significance	n	mantel	significance	n	mantel	significance	n	mantel	significance	n
REG	<NA>			0.62	1E-05	75	0.56	1E-05	75	0.60	1E-05	35
RE	0.62	1E-05	75	<NA>			0.72	1E-05	80	0.53	1E-05	36
RE.DUP	0.56	1E-05	75	0.72	1E-05	80	<NA>			0.50	1E-05	35
DUP	0.63	1E-05	56	0.53	1E-05	36	0.50	1E-05	35	<NA>		

Table S3: Mantel test results calculate the correlation among bacteria samples across different sampling strategies.
