# Supplementary material for: Microbial and metabolic succession on common building materials under high humidity conditions
Source: Nat Commun. 2019 Apr 16;10:1767. doi: 10.1038/s41467-019-09764-z (PMC6467912; doi:10.1038/s41467-019-09764-z)
Supplement: Supplementary file 3 — Reporting Summary [file 41467_2019_9764_MOESM3_ESM.pdf]

nature research

Corresponding author(s): Jack A Gilbert

Last updated by author(s): Mar 19, 2019

Reporting Summary

Nature Research wishes to improve the reproducibility of the work that we publish. This form provides structure for consistency and transparency in reporting. For further information on Nature Research policies, see Authors & Referees and the Editorial Policy Checklist.

Statistics

For all statistical analyses, confirm that the following items are present in the figure legend, table legend, main text, or Methods section.

n/a	Confirmed	
☐	☒	The exact sample size (n) for each experimental group/condition, given as a discrete number and unit of measurement
☐	☒	A statement on whether measurements were taken from distinct samples or whether the same sample was measured repeatedly
☐	☒	The statistical test(s) used AND whether they are one- or two-sided<br>*Only common tests should be described solely by name; describe more complex techniques in the Methods section.*
☐	☒	A description of all covariates tested
☐	☒	A description of any assumptions or corrections, such as tests of normality and adjustment for multiple comparisons
☐	☒	A full description of the statistical parameters including central tendency (e.g. means) or other basic estimates (e.g. regression coefficient) AND variation (e.g. standard deviation) or associated estimates of uncertainty (e.g. confidence intervals)
☐	☒	For null hypothesis testing, the test statistic (e.g. F, t, r) with confidence intervals, effect sizes, degrees of freedom and P value noted<br>*Give P values as exact values whenever suitable.*
☒	☐	For Bayesian analysis, information on the choice of priors and Markov chain Monte Carlo settings
☒	☐	For hierarchical and complex designs, identification of the appropriate level for tests and full reporting of outcomes
☐	☒	Estimates of effect sizes (e.g. Cohen's d, Pearson's r), indicating how they were calculated

Our web collection on statistics for biologists contains articles on many of the points above.

Software and code

Policy information about availability of computer code

Data collection	All sequencing technology used in the collection of this dataset is discussed in the methods.
Data analysis	R version 3.4.3; SparCC.py version Jun/20/2011

For manuscripts utilizing custom algorithms or software that are central to the research but not yet described in published literature, software must be made available to editors/reviewers. We strongly encourage code deposition in a community repository (e.g. GitHub). See the Nature Research guidelines for submitting code & software for further information.

Data

Policy information about availability of data

All manuscripts must include a data availability statement. This statement should provide the following information, where applicable:

- Accession codes, unique identifiers, or web links for publicly available datasets
- A list of figures that have associated raw data
- A description of any restrictions on data availability

The raw data from this project has been loaded to the QIITA database with study number 11950 as well as the European Nucleotid Archive from EMBL-EBI with study code PRJEB29658. OTU tables for the 16S and ITS datasets, the metabomomics dataset, the VLP and BLP counts, and the associated mapping files via FigShare at https://doi.org/10.6084/m9.figshare.7865015.v1 The code used for the analyses will be made available upon e-mail request to the authors.

Field-specific reporting

Please select the one below that is the best fit for your research. If you are not sure, read the appropriate sections before making your selection.

☐ Life sciences ☐ Behavioural & social sciences ☒ Ecological, evolutionary & environmental sciences

For a reference copy of the document with all sections, see nature.com/documents/nr-reporting-summary-flat.pdf

Ecological, evolutionary & environmental sciences study design

All studies must disclose on these points even when the disclosure is negative.

Study description	We used a factorial design to test the effect of material type, wetting, and inoculating location on the microbial community and metabolites associated with common building materials.
Research sample	Coupons of 4 building materials incubated over the course of 30 days
Sampling strategy	Sampling strategy is described in detail in the Methods section
Data collection	Data collection is described in detail in the Methods section
Timing and spatial scale	Samples were collected by swabbing the material coupons every 5 days for a period of 30 days.
Data exclusions	Certain samples were excluded from analysis because they did not produce enough reads to meet the rarefaction threshold. No samples were excluded at any point after the beginning of data analysis.
Reproducibility	Sampling replication of our longitudinal data was done via four sampling techniques (discussed in the text and in Figure S1) and Mantel tests were run to verify that patterns of beta-diversity were consistent across replicates
Randomization	All tiles used in this experiment were randomly assigned to experimental conditions, and were not believed to have any significant differences.
Blinding	Due to the complexity of the analysis, the number of people helping with the analysis, and the number of different variables included, blinding was difficult to implement, and we do not feel that blinding these results would have produced any significant different in the results beyond multi-hypothesis correction

Did the study involve field work? ☐ Yes ☒ No

Reporting for specific materials, systems and methods

We require information from authors about some types of materials, experimental systems and methods used in many studies. Here, indicate whether each material, system or method listed is relevant to your study. If you are not sure if a list item applies to your research, read the appropriate section before selecting a response.

Materials & experimental systems

n/a	Involved in the study	
☒	☐	Antibodies
☒	☐	Eukaryotic cell lines
☒	☐	Palaeontology
☒	☐	Animals and other organisms
☒	☐	Human research participants
☒	☐	Clinical data

Methods

n/a	Involved in the study	
☒	☐	ChIP-seq
☒	☐	Flow cytometry
☒	☐	MRI-based neuroimaging
